# Advertising Department

**Published:** 1874-12

**Authors:** 


*Advertising Department.*

To accomplish fully the object of a medical journal, its readers must be kept familiar with reliable manufacturers of chemicals and medicines, druggist's instruments, instrument makers, book merchants, and others engaged in business legitimately connected with the profession of medicine. For this purpose we propose to have a separate and independent advertising department. We will select none but first-class houses, therefore we ask the friends and readers of the RECORD to represent their claims for patronage.

